# Supplementary material for: MetaRibo-Seq measures translation in microbiomes
Source: Nat Commun. 2020 Jun 29;11:3268. doi: 10.1038/s41467-020-17081-z (PMC7324362; doi:10.1038/s41467-020-17081-z)
Supplement: Supplementary file 10 — Supplementary Data 7 [file 41467_2020_17081_MOESM10_ESM.zip › File2/Confidence_High_Taxonomy/320530_out.krona.html]

Javascript must be enabled to view this page.

members
magnitude
magnitudeUnassigned
count
unassigned
taxon
rank

320530\_out

2

2
superkingdom
2

1239
phylum
2

2
class
186801

186802
order
2

186803
2
family

species
1
39491

SRS013158\_contig\_number\_contig-100\_363.42869

1
genus
841


SRS098827\_contig\_number\_contig-100\_29999.29999
1262949
1
species
